# Myocardial Infarction in the Young: Aetiology, Emerging Risk Factors, and the Role of Novel Biomarkers

**DOI:** 10.3390/jcdd12040148

**Published:** 2025-04-10

**Authors:** Mithila Zaheen, Patrick Pender, Quan M. Dang, Eva Sinha, James J. H. Chong, Clara K. Chow, Sarah Zaman

**Affiliations:** 1 Westmead Applied Research Centre, Faculty of Medicine and Health, University of Sydney, Sydney, NSW 2145, Australiaclara.chow@sydney.edu.au (C.K.C.); 2 Department of Cardiology, Westmead Hospital, Sydney, NSW 2145, Australia; 3 Department of Cardiology, Liverpool Hospital, Sydney, NSW 2170, Australia; 4 Centre for Heart Research, Westmead Institute for Medical Research, University of Sydney, Westmead Hospital, Westmead, NSW 2145, Australia

**Keywords:** myocardial infarction, premature coronary disease, biomarkers

## Abstract

Despite significant advancements in the primary and secondary prevention of cardiovascular disease, evidence shows a rising incidence of premature coronary artery disease (CAD) and myocardial infarction (MI) in patients aged < 50 years. This increase is linked to the growing prevalence of traditional cardiovascular risk factors among younger people, such as type 2 diabetes, hypertension, obesity, and hyperlipidaemia, which have led to a rise in atherosclerotic CAD. Additionally, emerging research points to the influence of less traditional risk factors, including chronic inflammation, autoimmune diseases, drug use, psychosocial factors, and novel biomarkers in the early onset of CAD. These factors collectively contribute to the rise in premature CAD, highlighting the need for improved prevention strategies and public health efforts focused on younger populations. In this review, we explore the aetiology, risk factor profile, role of novel biomarkers, and how each of these impact outcomes among younger patients with MI.

## 1. Introduction

Coronary artery disease (CAD) and acute myocardial infarction (MI) are important causes of morbidity and mortality worldwide [1]. While the incidence of CAD is well known to increase with age, the proportion of acute MI events occurring in young patients is steadily increasing over time, particularly in young women [2,3,4]. This trend has sparked significant interest in understanding the underlying mechanisms of acute MI in the young, as premature CAD is associated with high rates of ischaemic recurrence, mortality, and public health impact due to premature mortality, morbidity, productivity loss, and healthcare costs [2,4].

Although there is no universally accepted definition of premature CAD, various studies have proposed age thresholds, typically ranging from 45 to 55 years, with some earlier studies using 65 years. More recently, an age cutoff of 49 years for men has been suggested [5]. Traditional cardiovascular risk factors, such as cigarette smoking and hyperlipidaemia, are implicated in the development of accelerated CAD [6]. In particular, a family history of premature atherosclerosis is associated with a younger age of MI [7]. The global rise in obesity and diabetes, now starting much earlier in life, is contributing to the growing burden of premature CAD [3,4,5].

The risk factor profile of young MI has evolved over time. Recent studies have identified a growing trend of ST-elevation myocardial infarction (STEMI) cases occurring in individuals without traditional modifiable risk factors [8]. Emerging research has underscored the increasing significance of non-traditional risk factors, including chronic inflammation, autoimmune diseases, hypercoagulability, and drug use, in the development of acute MI in younger populations [3,6]. Notably, non-atherosclerotic causes of acute MI are more prevalent in younger patients and are reported to account for up to 20% of cases [9]. Conditions like spontaneous coronary artery dissection (SCAD) and myocardial infarction with non-obstructive coronary arteries (MINOCA) are particularly common among younger people and especially women, with other non-atherosclerotic aetiologies such as coronary vasospasm and coronary vasculitis also prevalent causes of acute MI in younger populations [9].

In this review, we explore the aetiology, emerging risk factors, and novel biomarkers contributing to the development of premature CAD, providing insights into this growing public health concern.

## 2. Aetiology

### 2.1. Atherothrombotic MI

#### 2.1.1. Pathophysiology

Atherosclerotic CAD is the leading cause of acute MI in younger populations, with plaque rupture accounting for 60–65% of cases [5]. The pathophysiology of atherosclerosis involves a complex interplay of endothelial cell activation, monocyte infiltration, and the uptake of low-density lipoproteins (LDLs) by macrophages, leading to foam cell formation and local inflammation. This process is further influenced by immune responses, mediated by cytokines and lipid mediators, as well as haemodynamic forces acting on endothelial cells. These factors contribute to extracellular matrix remodelling and plaque development [10]. Over time, atherosclerotic plaques form a fibrous cap that can weaken due to mechanical stress and inflammation. When the plaque ruptures or erodes, it exposes thrombogenic material, triggering thrombus formation. In the coronary arteries, this thrombotic occlusion can block blood flow, resulting in ischaemia and acute MI, with the severity depending on the size and duration of the blockage [10]. While plaque rupture is recognised as the main cause of acute coronary syndrome (ACS), accounting for 60–75% of cases, plaque erosion and calcified nodules are also known to trigger blood coagulation cascades, resulting in ACS [11]. Despite considerable advancements, many aspects of the molecular mechanisms underlying plaque initiation and progression remain incompletely understood [10,12]. Previous autopsy studies have demonstrated distinct characteristics of coronary plaques in younger versus older patients with MI, suggesting that the underlying pathophysiological mechanisms driving the development of atherosclerosis and MI may differ between these age groups [13].

#### 2.1.2. Coronary Plaque Features in Premature CAD

Angiographic studies utilising intravascular imaging in patients with atherosclerotic CAD have revealed differing morphologies of culprit plaques across different age groups, although findings vary between studies. Younger patients with atherosclerosis generally present with a lower disease burden, characterised by fewer affected vessels, shorter lesions, and smaller plaque volumes [14]. A study by Xie et al. [15] describes that patients with premature CAD tend to have plaques with more fibrotic tissue, fewer necrotic or calcified components, and less frequent high-risk features, such as spotty calcification and cholesterol crystals. However, optical coherence tomography (OCT) studies present conflicting data regarding the characteristics of culprit plaques in different age groups. The OCT-FORMIDABLE is a European multicentre registry that found that younger patients (≤50 years) with STEMI were more likely to have plaque rupture, thinner caps, and fewer fibrotic or fibrocalcific components compared to older patients [16]. In contrast, a study by Fang et al. [17] that investigated culprit lesions with OCT revealed that younger patients (≤50 years) with STEMI had more plaque erosions and fewer thin-cap fibroatheromas. This study suggests that, despite having lower plaque burden and calcification, younger patients are more prone to plaque rupture and thrombus formation, indicating greater plaque instability [17,18]. Additionally, sex differences have been observed, with young men having larger plaque volumes and more nonculprit lesions, while young women tend to have thicker fibrous caps and less plaque calcification, suggesting the role of oestrogen in stabilising fibrous caps [14]. Similarly, a 2022 study by Seegers et al. [19] examined age- and sex-related differences in plaque characteristics among 1368 ACS patients using OCT imaging. There was evidence of increasing rates of plaque rupture with age. Younger women (≤50 years) showed a trend towards a higher prevalence of plaque erosion compared to men of the same age (74% vs. 53%, *p* = 0.08), while both sexes experienced a similar decline in plaque erosion with ageing (27% in women vs. 32% in men >80 years). The higher prevalence of plaque erosion in young women further supports the hypothesis that oestrogen may have a protective, anti-inflammatory effect against plaque rupture but not erosion [19]. Overall, while younger patients tend to have less advanced atherosclerosis, they appear to be at a higher risk for plaque instability [14,16,17,20]. Further research is necessary to better understand the age-related variations in plaque characteristics and progression.

### 2.2. Myocardial Infarction with Non-Obstructive Coronary Arteries

ACS is typically caused by coronary artery obstruction due to atherosclerotic plaque growth or thrombus formation resulting from plaque rupture or erosion. However, some patients exhibit signs and symptoms of myocardial infarction without significant coronary artery obstruction, a condition known as MINOCA [21]. The prevalence of MINOCA ranges from 5 to 25%, with the highest incidence observed in patients ≤ 35 years old (over 18%) [21]. However, MINOCA remains a working diagnosis and should prompt investigation to look for the underlying cause. In some cases of MINOCA, an alternate non-ischaemic cause is found (such as myocarditis or Takosubo’s syndrome), where myocardial infarction is not present, and the working diagnosis of MINOCA no longer applies. The risk profile of MINOCA patients is similar to that of those with obstructive CAD, though MINOCA patients show higher levels of drug use and depression, and a higher proportion are females [21]. Additionally, higher triglyceride levels have been noted, though the difference compared to other groups is only borderline significant [21].

The VIRGO (Variation in Recovery: Role of Gender on Outcomes of Young AMI Patients) registry, which enrolled young patients (18–55 years) with acute MI between 2008 and 2012, used angiography-based classification to divide the cohort into those with significant obstruction (plaque ≥ 50%, 88.4%), MINOCA (plaque < 50%, 11.1%), or other aetiologies [22]. Women in this study had almost five times the incidence of MINOCA compared to men. A large majority (75%) of MINOCA cases remained undefined, and a minority were categorised as SCAD, coronary spasm, or embolisation—which will be separately discussed in this review [22]. These findings underscore the need for a systematic approach and extensive use of imaging (intravascular, echocardiography, and cardiac magnetic resonance imaging) to better characterise and understand the diverse mechanisms underlying MINOCA.

### 2.3. Non-Atherosclerotic Coronary-Related MI

#### 2.3.1. Spontaneous Coronary Artery Dissection

Spontaneous coronary artery dissection (SCAD) is a condition characterised by the spontaneous separation of the coronary artery wall, unrelated to trauma, iatrogenic causes, or atherosclerosis. This separation can lead to coronary obstruction due to an intimal tear or the formation of an intramural haematoma [23,24]. SCAD has several known precipitating or associated factors, including fibromuscular dysplasia, pregnancy, multiparity, hormonal therapy, connective tissue disorders, and systemic inflammatory diseases [25]. The condition predominantly affects women, who account for approximately 80–90% of cases, with a mean age of presentation between 44 and 62 years. SCAD accounts for about 35% of acute coronary syndrome cases in women under 50 [24,25,26]. Coronary angiography is the gold standard for diagnosis, though a high degree of suspicion is required, as the typical angiographic findings—extraluminal contrast staining with multiple radiolucent lumens—are observed in only a minority of cases [24,25]. Intravascular imaging, such as OCT or intravascular ultrasound (IVUS), can aid the diagnosis, but these tools must be cautiously used in the presence of a false lumen [27]. Evidence-based management of SCAD remains challenging given the limited data to guide treatment.

#### 2.3.2. Coronary Vasospasm

Coronary vasospasm is a rare but significant cause of acute MI in young patients and may initially fall under the MINOCA working diagnosis [23,28]. It is characterised by transient, severe coronary artery occlusion (greater than 90% constriction), which results in angina and ischaemic electrocardiographic changes, either spontaneously or triggered by stimuli such as acetylcholine, ergotamine, or hyperventilation [29,30]. Coronary vasospasm accounts for about 20% of MINOCA cases in young patients, with a higher prevalence observed in East Asians [28].

Although the exact prevalence is unclear, coronary vasospasm primarily affects younger patients, with no significant gender disparity. The pathophysiology is multifactorial, involving autonomic overstimulation, endothelial dysfunction, oxidative stress, and smooth muscle hypercontractility. Provocative testing, such as intracoronary acetylcholine or ergonovine administration, may be required to confirm the diagnosis since the vasospasm often resolves before coronary angiography. Smoking and illicit drug use are key triggers for vasospasm [28].

Smoking cessation is crucial for improving prognosis, followed by avoidance of vasospasm-inducing medications and medical treatment with calcium channel blockers and nitrates [30]. Despite treatment, vasospasm may recur in 4–19% of cases. With appropriate therapy and lifestyle modifications, the prognosis is generally favourable [29]. Dedicated studies on coronary vasospasm in young patients with acute MI are lacking.

#### 2.3.3. Coronary Embolism and Hypercoagulability

Coronary embolism and thrombosis are known to contribute to acute MI in young patients, particularly those with hypercoagulable disorders [31]. These conditions lead to abnormal activation of the coagulation cascade, thereby increasing the propensity for thrombus formation [31]. Key causes of coronary embolism include atrial fibrillation, in which thrombi form in the atria and embolise to the coronary circulation, as well as cardiomyopathy, infective endocarditis, valvular heart disease, malignancy, and systemic autoimmune diseases [32]. Among these, antiphospholipid syndrome, characterised by the presence of antiphospholipid antibodies, is a well-established risk factor, predisposing individuals to both acute coronary thrombosis and accelerated atherosclerotic progression [33]. Hereditary thrombophilias, including protein C/S deficiency, antithrombin deficiency, Factor V Leiden mutation, and hyperhomocysteinaemia, have been linked to an increased risk of coronary artery embolism [34].

Paradoxical coronary thromboembolism through a patent foramen ovale (PFO) is a rare mechanism where a venous thrombus bypasses the lungs via a right-to-left shunt, entering systemic circulation and potentially causing coronary artery occlusion. While pulmonary embolism is the typical outcome of venous thrombosis (e.g., deep vein thrombosis), paradoxical embolism through a PFO more commonly affects the cerebral circulation due to its anatomical position. However, other arterial beds, including the coronary arteries, can also be involved. Paradoxical coronary embolism accounts for 10–15% of all paradoxical embolic events and less than 1% of acute myocardial infarctions, though it may contribute to up to 25% of acute coronary events in patients under 35 years old [34,35].

Coronary embolism and thrombosis can also manifest as MINOCA, particularly when small coronary vessels are implicated or when spontaneous thrombolysis occurs [23,32]. In the VIRGO study, coronary artery embolism was identified in 4% of young MI patients and in 1% of those with MINOCA [22]. Management of these conditions involves addressing the underlying hypercoagulable state, utilising anticoagulant or antiplatelet therapy to mitigate thrombus formation, and providing specific treatment for any concurrent conditions such as atrial fibrillation or infective endocarditis [31].

#### 2.3.4. Coronary Artery Vasculitis

Coronary artery vasculitis (CAV) is an important differential diagnosis to consider in younger patients with unexplained ACS or congestive heart failure, particularly in the context of known primary or secondary vasculitis [36]. These conditions may present as the primary manifestations in younger individuals without traditional cardiovascular risk factors, such as those identified in the Framingham risk score [37]. However, the global epidemiology of CAV is not well understood due to factors such as underdiagnosis, low incidence rates, and variability in the sensitivity and specificity of current diagnostic tools [36].

CAV can present with symptoms similar to those of typical CAD, including angina, acute MI, arrhythmias, conduction disturbances, or heart failure [37]. The overproduction of pro-inflammatory cytokines is thought to contribute to the inflammatory changes in the smooth muscle walls of the coronary arteries, which underlie the pathogenesis of CAV [37].

CAV can be seen in various diseases such as Kawasaki disease (KD), Takayasu arteritis (TA), polyarteritis nodosa (PAN), and giant cell arteritis (GCA). Primary medium-vessel vasculitides like PAN (incidence rate of 4–10 per million) and KD (incidence rate of 2 per million) can involve the coronary arteries in up to 50% and 20% of cases, respectively [38,39]. Large vessel vasculitides like TA and GCA have lower incidences of coronary involvement, with estimates of 10–30% and <1%, respectively [37]. Each CAV syndrome presents with distinct noncoronary vasculitis features. The management of CAV is primarily focused on treating the underlying autoimmune disease to reduce the degree of cardiac involvement [36].

**Figure 1:**
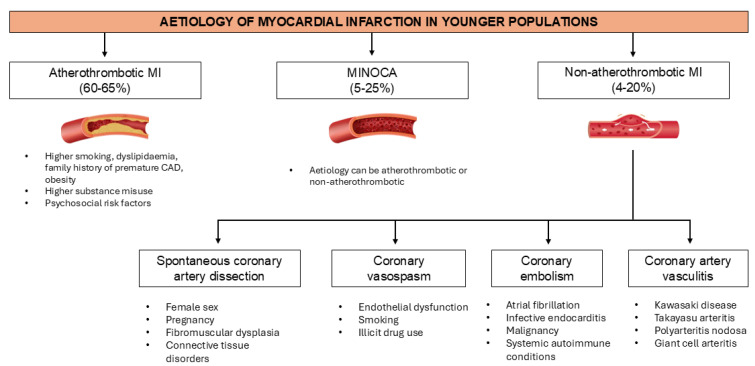
Causes of myocardial infarction in younger populations and associated risk factors.

Figure 1 provides a summary of the common aetiologies of myocardial infarction in young individuals and associated risk factors.

## 3. Risk Factors for Acute Myocardial Infarction in Younger Populations

### 3.1. Traditional Cardiovascular Risk Factors

The primary cause of acute MI in younger populations is atherosclerotic plaque rupture, which is associated with traditional cardiovascular risk factors such as dyslipidaemia, hypertension, diabetes, smoking, obesity, and a family history of CAD [14,40]. However, the distribution of these risk factors differs between younger and older populations [41]. Global acute MI registries consistently show that smoking, dyslipidaemia, and a family history of premature CAD are more prominent in younger patients. In contrast, diabetes, metabolic syndrome, and hypertension are more common in older populations [42,43,44,45,46]. Furthermore, patients with premature CAD often have poorer control of traditional modifiable risk factors and are less likely than older counterparts to meet blood pressure and low-density lipoprotein (LDL) targets [47]. One possibility is that healthcare providers may be less aggressive in managing cardiovascular risk in younger patients, perhaps due to a perception of lower immediate risk or concerns about long-term medication adherence. Additionally, younger individuals with premature CAD may have more severe underlying metabolic abnormalities, such as treatment-resistant hypertension, familial hypercholesterolaemia, or significant obesity, which can render standard guideline-based therapies insufficient to achieve optimal control. Furthermore, lifestyle-related factors, including lower adherence to dietary modifications, physical activity recommendations, and medication regimens, may further hinder effective risk factor management in this population [47].

#### 3.1.1. Smoking

Smoking remains the most significant and prevalent risk factor for young MI patients. A large STEMI registry by Zasada et al. [6], encompassing over 230,000 patients, revealed that individuals under 40 years of age had a significantly higher proportion of current smokers compared to older counterparts (37.5% vs. 23.0%; *p* < 0.001) [6]. Several smaller-scale studies support this finding, with smoking rates reported to be as high as 71.2% in Mahendiran et al.’s [48] 20-year analysis of ACS patients under 50 in Switzerland and 72.1% in patients aged ≤ 45 years in Lv et al.’s [49] acute MI registry in China, consisting of over 24,000 patients.

Notably, smoking not only increases the risk of MI but also the likelihood of recurrent events [41]. Research from the Partners YOUNG-MI registry highlights that smoking cessation is the most effective intervention for improving long-term outcomes, reducing all-cause and cardiovascular mortality by over 50% [5,50]. Concerningly, the EuroAspire IV cross-sectional study of 7998 patients post-ACS or coronary intervention demonstrated that young patients are more likely to continue smoking post-coronary event [51]. These findings underscore the critical importance of early intervention to address modifiable risk factors and promote healthier lifestyle choices in younger patients presenting with MI.

#### 3.1.2. Lipid Disorders

Dyslipidaemia is a well-established risk factor for MI across all age groups. Over half of patients with premature CAD have familial lipoprotein disorders [5,52]. Around 10–20% of these patients are diagnosed with heterozygous familial hypercholesterolaemia (FH), and the prevalence of familial combined hyperlipidaemia ranges from 12.5% to 16% [5]. A study by Bogsruf et al. [53], which examined 328 patients with acute MI, found that the prevalence of FH in young patients was higher than in the general population, yet it often went undiagnosed using the Dutch Lipid Clinic Network score for clinical FH. This suggests that increasing genetic screening for FH in young MI patients could help identify more cases, allowing for early intervention and providing an opportunity to treat affected family members.

#### 3.1.3. Obesity

The risk profile of young MI patients is evolving, with obesity emerging as a significant and increasingly prevalent factor. Multiple studies highlight this shift, with Matsis et al. [54] reporting that 36% of young MI patients ≤ 50 years in their cohort had none or only one traditional risk factor, classifying them as low risk prior to their event, yet obesity was notably common in this group (50% vs. 36.6%, *p* < 0.001). Similarly, Mahendiran et al. [48] observed a substantial rise in young MI patients with obesity over 20 years (from 21.2% to 27.1%, Ptrend < 0.001) and that patients aged <50 years old were more likely to be obese (21.7% vs. 17.4%, *p* < 0.001). Supporting this trend, King et al. [55] demonstrated that obesity rates were the highest in those aged <50 years (20.4%, *p* < 0.001) in a New York STEMI registry of over 580,000 patients, emphasising the growing importance of addressing obesity in the prevention and management of MI in younger individuals.

### 3.2. Non-Traditional Risk Factors

#### 3.2.1. Substance Misuse

Substance misuse is a significant risk factor for MI in young patients, with minimal prevalence in older populations [56]. Recreational drug use, including cocaine and cannabis, has been reported in nearly 25% of young ACS patients, often associated with larger infarctions and poorer left ventricular function compared to non-users [57]. Cocaine, in particular, is a well-established risk factor for ACS, exerting both acute vasoconstrictive and chronic atherogenic effects that accelerate coronary atherosclerosis [56]. Gresnigt et al. [57] highlighted the prevalence of cannabis (16.2%), cocaine (4.8%), and combined use (2.6%) among young ACS patients. Similar findings were noted in the YOUNG-MI cohort, with very young patients (defined as age ≤ 40 years) demonstrating greater substance misuse compared to those aged ≤ 50 years (17.9% vs. 9.3%; *p* < 0.001) [41]. Alcohol misuse is also prevalent, with studies from STEMI registries in India and China showing high rates of alcohol intake and alcohol use disorders, up to 67.8%, among young MI patients [43,58]. However, data from a national analysis on cardiovascular disease burden in young Black patients in the USA by Vyas et al. [59] suggest a declining trend in substance and alcohol misuse over the past decade. Despite this, the persistent association between substance misuse and young MI underscores the need for targeted prevention and intervention strategies.

#### 3.2.2. Chronic Inflammation and Autoimmune Diseases

Atherosclerosis is in part a chronic inflammatory process. Monocyte infiltration of the endothelium and subsequent macrophage transformation leads to the release of pro-inflammatory cytokines and further recruitment and activation of immune cells [60]. Consequently, inflammatory markers such as high-sensitivity CRP (hs-CRP), tumour necrosis factor, and interleukins (ILs) have been shown to be associated with increased risk of atherosclerosis and cardiovascular events [61]. This is true also in young MI, with a recent study by Cederström et al. [62] demonstrating that IL-6 and IL-8 could act as possible biomarkers for long-term cardiovascular events following acute MI in young patients. This raises the possibility that markers of a chronic inflammatory state could also be used to screen for patients at risk of premature MI, as will be discussed later in this review. Therapies targeting inflammatory processes have been trialled in the general population post-MI, in particular colchicine and canakinumab, an IL-1β monoclonal antibody [60]. These novel treatments have demonstrated significant reduction in further cardiovascular events and mortality, and dedicated studies to investigate their role in young MI are warranted.

Autoimmune diseases are also emerging as novel risk factors for young patients with acute MI. Weber et al. [63] reported that 2.5% of young MI patients had systemic inflammatory disease (SID), which was associated with higher rates of long-term mortality. Systemic lupus erythematosus (SLE), in particular, is linked to systemic and endothelial inflammation, accelerating atherosclerosis and increasing the risk of MI [64]. Studies show that up to 16% of SLE patients may experience MI, and those aged 35 to 44 are nearly 50 times more likely to suffer an MI compared to healthy controls, as noted in the Framingham Offspring study [64]. Psoriasis, a common T-helper cell type 1 autoimmune disease, is also associated with an increased risk of MI, with young patients suffering from severe disease disproportionately impacted [65]. A 2006 cohort study of 687,971 patients demonstrated that a 30-year-old patient with severe psoriasis faced an adjusted relative risk of MI of 3.10 versus 1.36 for a 60-year-old patient [66]. Currently, biologic therapies are being investigated as a means of treating cutaneous disease while simultaneously reducing systemic inflammation and subsequent cardiovascular risk [65].

Another evolving area is the complex relationship between oral health conditions and cardiovascular disease, in particular periodontitis. Individuals with periodontitis are known to have an increased risk of cardiovascular disease [67]. Oral pathogens associated with periodontitis have been shown to induce endothelial dysfunction and accelerate atherosclerosis in animal models [67]. The theorised pathophysiology is that oral bacteria trigger local pro-inflammatory cytokine cascades, precipitating a chronic inflammatory state. While the incidence of periodontitis increases with age, up to 30% of all adults suffer from moderate or severe disease [68]. Thus, further investigation into whether treatment of periodontitis and other oral health conditions improves cardiovascular outcomes could also reduce the incidence of young MI.

### 3.3. Female-Specific Risk Factors

Women are more likely to experience premature MI than men [3]. Young women with MI have distinct risk factors and pathophysiological mechanisms compared to men and older women [22]. They are more likely to experience coronary microvascular dysfunction, SCAD, and plaque erosion rather than rupture [69]. Studies show that young women tend to recover more slowly and are at higher risk for morbidity, mortality, and readmission compared to their age-matched male counterparts [70,71]. Of note, women of all ages are less likely than their male counterparts to receive coronary angiography or revascularisation, be prescribed guideline-directed secondary prevention, and be referred to cardiac rehabilitation post-ACS [72].

Traditional cardiovascular risk factors do not account for the total observed burden of CAD in women [71,73]. Pregnancy-related complications are a significant contributor to cardiovascular disease risk in young women. Conditions such as pre-eclampsia, gestational diabetes, gestational hypertension, preterm delivery, and low-birth-weight infants are linked to endothelial dysfunction, metabolic disturbances, and premature atherosclerosis [69,74]. A multitude of studies have demonstrated that women with these risk factors are at significantly higher risk of developing CAD, and this has resulted in pre-eclampsia being recognised as a female-specific cardiovascular risk factor by the American Heart Association [74]. In fact, a 2022 cross-sectional cohort study demonstrated that women with pregnancy-related cardiometabolic conditions were at greater risk of severe cardiovascular outcomes within two months of pregnancy, although this included additional diagnoses such as heart failure and pulmonary embolism [75]. Recurrent miscarriage and placenta-mediated complications have also been shown to further increase the likelihood of premature CAD [76]. Furthermore, women diagnosed with gestational diabetes or hypertensive disorders of pregnancy are inadequately followed up postpartum to manage their ongoing cardiovascular risk [77]. A 2020 systematic review by Jones et al. [78] reported less than 58% of women with gestational diabetes were screened for subsequent diabetes mellitus within four months of delivery.

Hormonal factors also play a role in premature CAD risk. A large multicentre observational study by Manzo-Silverman et al. [79] of women aged < 50 years with MI in France highlighted that these patients experienced a high prevalence of adverse pregnancy outcomes (31.2%) and prescription of combined oral contraceptives despite the patient having at least one contraindication (86.0%). The effect of prolonged oestrogen exposure from early menarche has been studied, but to date this has not been definitively associated with a higher risk of developing premature CAD [80,81].

### 3.4. Psychosocial Risk Factors

Emerging psychosocial risk factors are increasingly recognised in young patients with premature MI, with studies highlighting the significant role of anxiety, depression, and socioeconomic factors [82]. In the GENESIS-PRAXY study, investigating patients < 55 years with ACS, young women exhibited a greater prevalence of non-traditional risk factors such as anxiety, low household income, and depression compared to their male counterparts [73].

Socioeconomic status, including low income and low education levels, has also been identified as a key risk factor for premature MI [83,84]. A U.S. study projected that individuals with low socioeconomic status were twice as likely to develop acute MI by the age of 65 compared to those with higher socioeconomic status [84]. Furthermore, rural populations appear to be particularly vulnerable to these risks [84]. Education level has also been shown to independently predict two-year mortality rates in young patients aged < 45 years with acute MI, as demonstrated in a study in China by Lv et al. [49].

Additionally, there is growing recognition of the prevalence and impact of psychiatric disorders in young patients with MI. King et al. [55] described that major psychiatric illness was more common in patients aged < 50 years compared to the older population. Moreover, Vyas et al. [59] highlight the increasing burden of depression in young Black patients with CAD in 2017 versus 2007 (6.8 vs. 4.0, *p* < 0.001).

Emerging lifestyle factors, such as increased television viewing, have also been linked to higher cardiovascular risk. A study from the Coronary Artery Risk Development in Young Adults (CARDIA) cohort showed that each additional hour of daily television viewing was associated with a higher risk of coronary heart disease, stroke, and other cardiovascular events [85]. These psychosocial and lifestyle factors underscore the need for a holistic approach to preventing premature cardiovascular events in young populations.

### 3.5. Environmental Risk Factors

There is growing recognition of the importance of the environment in the development of cardiovascular disease. Factors such as pollution, toxin exposure, the built environment, food availability, and socio-cultural influences have all been implicated in shaping cardiovascular risk [86]. Addressing these broader influences is essential for effective population-level prevention strategies. Air pollution, such as particulate matter (PM), has been linked to several adverse cardiovascular outcomes, including heart failure, ischaemic heart disease, arrhythmia, and peripheral vascular disease [87,88]. A study on the genetics of atherosclerotic disease by Posadas-Sanchez et al. has shown that ozone exposure at different time points and PM exposure at 5 years were associated with increased odds of developing premature CAD [89]. Microplastics and nanoplastics are emerging as a potential risk factor for cardiovascular disease in preclinical studies [90,91]. However, while the potential harms are becoming clearer, there is no definitive evidence yet to establish a direct link to premature CAD [91]. Further research is needed to fully understand the impact of these environmental factors on cardiovascular health.

Table 1 summarises global studies on young myocardial infarction, highlighting the burden of both traditional and non-traditional risk factors.

## 4. Role of Novel Biomarkers

### 4.1. Lipoprotein(a)

Lipoprotein(a) [Lp(a)] is emerging as a novel biomarker for the development of premature CAD and acute MI in younger patients [95]. Elevated Lp(a) levels are strongly linked to an increased risk of CAD and ischaemic stroke, with the risk being particularly pronounced in those under 45 years [5]. Studies show that Lp(a) levels above 50 mg/dL are associated with a three-fold higher likelihood of ACS in younger individuals, and this association diminishes with age [96]. The Mass General Brigham YOUNG-MI registry found that one-third of patients aged 50 or younger with acute MI had Lp(a) levels greater than 50 mg/dL [97]. Additional small-scale studies have highlighted the strong association between elevated Lp(a) levels and premature CAD, with up to 32% of young MI patients showing elevated levels [98,99]. Higher Lp(a) levels have also been linked to the presence of multivessel coronary lesions in young MI patients [100].

### 4.2. High-Sensitivity C-Reactive Protein

The role of high-sensitivity C-reactive protein (hs-CRP) as a biomarker for premature MI remains an area of investigation. Early studies suggest that hs-CRP may help predict future coronary events in young patients with a history of acute coronary syndromes, particularly when plasma levels exceed 1.6 mg/L [101]. More recently, a 2024 study by Song et al. [102] indicated that hs-CRP, along with the atherogenic index of plasma (AIP), served as an independent risk factor for premature CAD, with higher hs-CRP levels correlating with greater disease severity. Liu et al. [103] highlight the value of CRP in assessing risk among young women with ACS, suggesting its utility in risk stratification. However, further research is needed to clarify hs-CRP’s definitive role and its utility in predicting premature MI.

### 4.3. Apolipoproteins

The use of apolipoproteins as biomarkers for young MI remains poorly established due to the limited and low-quality evidence available. A study by Zhang et al. [104] found that among a young STEMI cohort of patients aged ≤40 years, serum apolipoprotein A was a prognostic marker and was shown to be associated with the risk of heart failure and malignant arrhythmias. A prospective cohort study using the UK Biobank by Martson et al. [105] concluded that the risk of MI was best captured by apolipoprotein B-containing lipoproteins, independent from lipid content. This suggests that apolipoprotein B may be a key factor in the development of atherosclerosis, but these findings were not specific to young MI patients [105]. A systematic review by Aghajani et al. [106] indicated a possible link between apolipoprotein A-1 levels and premature CAD but noted that the studies included were mostly case–control designs, which limits the strength and reliability of the conclusions. As such, the current evidence is insufficient, and further high-quality prospective cohort studies are needed to determine the value of apolipoproteins as biomarkers for young MI.

### 4.4. Homocysteine

The role of homocysteine (Hcy) as a biomarker for premature CAD and young MI has been supported by a few small studies, though the evidence is still limited. Schaffer et al. [107] reported that elevated plasma Hcy levels were independently associated with CAD. A small case–control study by Sadeghian et al. [108] found that hyperhomocysteinaemia was a risk factor for CAD in young patients aged <45 years, particularly in men, and that vitamin B12 deficiency may contribute to elevated Hcy levels. Barghash et al. [109] identified a significant link between elevated Hcy and low folate levels with premature CAD, suggesting that Hcy, along with folate and plasminogen activator inhibitor-1, may serve as independent risk factors. While these studies point to the potential of Hcy as a marker for early cardiovascular risk, the evidence is based on small sample sizes and case–control designs, indicating the need for larger, more robust studies to confirm its role in young CAD patients.

### 4.5. Gut Microbiome

The gut microbiome may play an emerging role in the pathogenesis of MI in younger populations. Dysbiosis, or disturbances in the gut microbiome, has been linked to cardiovascular diseases, including coronary artery disease (CAD) [110]. Studies suggest that microbiome-derived metabolites like trimethylamine N-oxide (TMAO), produced by bacteria such as Bacteroides, contribute to atherosclerosis and CAD [111,112]. A case–control study by Liu et al. [113] found significant differences in gut microbiome diversity between young patients aged <44 years with STEMI and those without CAD, with alterations in the abundance of specific bacteria like Streptococcus and Prevotella. Notably, an inverse relationship was observed between Prevotella and obesity, with a decrease in Prevotella associated with higher inflammatory markers in obese patients [113]. These findings highlight the potential for integrating gut microbiome analysis with clinical parameters to enhance diagnostic efficacy and better understand the mechanisms underlying premature CAD in young patients.

## 5. Impact of Risk Factors on Outcomes in Young MI

The burden of traditional and non-traditional risk factors plays a crucial role in determining long-term outcomes, with a significant proportion of these factors being modifiable. Studies have shown that over 90% of young MI patients had at least one modifiable risk factor at the time of presentation, and approximately 60% had two or more [92]. Research from the Partners YOUNG-MI registry emphasises that smoking cessation can improve long-term outcomes, reducing both all-cause and cardiovascular mortality by over 50% [50]. These findings underscore the importance of early intervention to address modifiable risk factors, particularly smoking, in younger MI patients. Psychosocial factors also influence outcomes, with education levels shown to independently predict two-year mortality rates in young patients aged <45 years with acute MI, as demonstrated in a study in China by Lv et al. [49].

Overall, the majority of young MI registries worldwide report a favourable prognosis with a better overall morbidity and mortality rate compared to older cohorts [43,44,45,46,55]. However, one study by Ando et al. reported that younger individuals with STEMI were more prone to presenting with cardiopulmonary arrest, which was strongly associated with in-hospital mortality [42]. A U.S. study by Shamaki et al. [93] of 41,990 young STEMI patients (aged 18–45) found that those without standard modifiable risk factors (SMuRF-less) had significantly worse in-hospital outcomes compared to their counterparts with such risk factors. SMuRF-less patients exhibited higher adjusted in-hospital mortality (aOR 2.6), cardiogenic shock (aOR 1.8), acute kidney injury (aOR 1.4), and the need for extracorporeal membrane oxygenation (aOR 4.1), underscoring the severity of their clinical presentations despite the absence of conventional risk factors.

Sex differences emerge as a critical factor in the outcomes of young patients with acute MI. Studies have consistently found that young women have worse clinical outcomes than men [22,43,46,49]. It has been reported that young women have longer symptom onset-to-admission times, were less likely to receive percutaneous coronary intervention (PCI), and experienced higher mortality than young men [114]. Furthermore, the VIRGO study found that young women experience more adverse outcomes in the year after discharge, particularly in terms of non-cardiac hospitalisations [22]. This may be partly related to sex differences in risk factor burden, with studies demonstrating young women had higher numbers of cardiovascular risk factors compared to young men [22,46]. Women aged < 35 years have been shown to have a higher risk of revascularisation and recurrent acute MI [94]. These findings underscore the need for gender-specific strategies in the management and prevention of acute MI in young patients.

## 6. Public Health Implications and Future Directions

The increasing prevalence of MI among young individuals poses a significant public health challenge, marked by unique clinical profiles, non-traditional risk factors, and pronounced disparities in outcomes, particularly for young women. The societal and economic burden of premature cardiovascular events is large. Myocardial infarction at a young age leads to a substantial loss of quality-adjusted life years, affecting the individual’s long-term health and productivity, with a long-term burden on the healthcare system [2]. This underscores the urgency of developing improved strategies for diagnosis, risk stratification, and management. Since atherosclerotic disease remains the most common cause of young MI, prevention efforts must focus on addressing modifiable risk factors such as smoking, dyslipidaemia, and obesity [5]. Public health strategies should also incorporate education on the role of emerging contributors, including psychosocial factors, to better combat premature CAD. Further research is needed to validate the use of novel diagnostic tools in risk prediction, including biomarkers like hs-CRP, lipoprotein(a), apolipoproteins, and homocysteine, and to explore the potential roles of genetic screening, environmental pollutants, and gut microbiome dysbiosis in young MI.

Equally critical are targeted interventions to address disparities in care and outcomes, particularly for young women. Women with MI often face delays in care, less intensive treatment, and worse post-MI outcomes compared to men [72]. These challenges are compounded during vulnerable periods such as pregnancy and the peripartum period, emphasising the need for gender-specific research and tailored strategies. Addressing these disparities requires not only clinical efforts but also systemic changes to improve access to timely and equitable care, ultimately enhancing outcomes for young women with MI.

## 7. Conclusions

The growing burden of MI in younger populations poses a critical public health challenge, as its aetiology is multifaceted, involving both atherosclerotic and non-atherosclerotic causes. Alongside the increasing burden of traditional cardiovascular risk factors, the influence of non-traditional risk factors, psychosocial factors, and novel biomarkers in the development of premature CAD is becoming increasingly evident. Effective public health strategies must prioritise the prevention of modifiable risk factors such as smoking, obesity, and dyslipidaemia while also addressing emerging contributors like environmental stressors and psychosocial factors. Furthermore, gender-specific interventions are needed to bridge outcome gaps, particularly for young women, who have poorer outcomes compared to their young male counterparts.

## Figures and Tables

**Table 1:** Summary table of studies on risk factors in young myocardial infarction patients.

Authors	Country	Aim of Study	Study Type	Sample Size	Study Population	Traditional Cardiovascular Risk Factors	Non-Traditional Risk Factors
Matsis, Holley, Al-Sinan et al., 2017 [54]	New Zealand	Compare clinical characteristics in young and older MI patients	Retrospective observational study	Total: 1199 Age ≤ 50 years: 154	≤50 years	Hypertension (35.7%) Diabetes (13.6%) Dyslipidaemia (57.1%) Previous MI (10.4%) Family history of premature CAD (48.7%) Former smoker (23.4%) Current smoker (47.4%) Obesity (50.0%)	Not reported
Yandrapalli, Nabors, Goyal et al., 2019 [92]	USA	Investigate the clinical characteristics of young MI patients	Retrospective cohort study	Total: 1,462,168 Age < 44 years: 280,875 Age 45–59 years: 1,181,282	<45 years	Hypertension (49.8%) Diabetes (22.6%) Dyslipidaemia (51.7%) Smoker (56.8%) Obesity (20.7%)	Illicit drug use (9.6%)
Zeitouni, Clare, Chiswell et al., 2020 [4]	USA	Compare risk factor burden and long term prognosis of patients with premature CAD	Retrospective and prospective observational study	Total: 101,061 Age < 50 years: 3655 (3.6%)	<50 years	Hypertension (52.8%) Diabetes (23.8%) Dyslipidaemia (46.4%) Family history of CAD (39.8%) Former smoker (11.4%) Current smoker (49.4%) Obesity (47.1%)	Connective tissue disease (0.7%) History of cancer (5.3%) Chronic inflammatory disease (2.3%)
Yang, Biery, Singh et al., 2020 [41]	USA	Investigate risk factors and outcomes of young MI patients	Retrospective observational study	Total: 2097 Age ≤ 40 years: 431	≤40 years	Hypertension (37.9%) Diabetes (18.8%) Dyslipidaemia (90.7%) Family history of premature CAD (31.3%) Current smoker (52.2%) Obesity (36.9%)	Alcohol use (10.1%) Illicit substance use (17.9%)
Lv, Ni, Liu et al., 2021 [49]	China	Compare clinical characteristics, prognosis, and gender disparities in young MI patients	Retrospective observational study (registry)	Total: 24,125 Age ≤ 45 years: 2042	≤45 years	Hypertension (33.5%) Diabetes (11.3%) Dyslipidaemia (10.4) Previous MI (4.5%) Family history of premature CAD (7.1%) Current smoker (72.1%) Obesity (18.3%)	Not reported
Zasada, Bobrowska, Plens et al., 2021 [6]	Poland	Compare differences in clinical characteristics and treatment strategies between young and older patients with acute MI	Retrospective observational study (registry)	Total: 237,747 Age < 40 years: 3208 (1.3%)	<40 years	Hypertension (29.96%) Diabetes (5.33%) Previous MI (7.17%) Smoker (37.5%)	Not reported
Ando, Yamaji, Kohsaka et al., 2022 [42]	Japan	Investigate clinical and angiographic characteristics of young MI patients who underwent PCI	Retrospective cohort study	Total: 213,297 Age < 50 years: 23,985	<50 years	Hypertension (54.1%) Diabetes (30.4%) Dyslipidaemia (65.5%) Previous MI (10.4%) Current smoker (62.8%)	Not reported
Khraishah, Karout, Jeong et al., 2022 [46]	India	Compare clinical characteristics and outcomes in young MI patients	Retrospective observational study (registry)	Total: 21,374 Age ≤ 50 years: 4762	≤50 years	Hypertension (33.8%) Diabetes (35.7%) Smoker (41.9%) Obesity reported to be more prevalent in young population	Not reported
Mahendiran, Hoepli, Foster-Witassek et al., 2023 [48]	Switzerland	Compare trends in cardiovascular risk factors between young and older MI patients	Retrospective observational study (registry)	Total: 58,028 Age < 50 years: 7073 (14.1%)	<50 years	Hypertension (35.9%) Diabetes (10.1%) Dyslipidaemia (57.3%) Family history of CAD (43.1%) Previous MI (9.2%) Former smoker (12.6%) Current smoker (71.4%) Obesity (21.7%)	Not reported
Kumar, Ammar, Qayyum et al., 2023 [45]	Pakistan	Compare clinical characteristics, angiographic patterns, and outcomes of young patients with STEMI	Retrospective observational study	Total: 4686 Age ≤ 40 years: 466	≤40 years	Hypertension (53.1%) Diabetes (35.4%) Metabolic syndrome (23.1%) Previous MI (8.9%) Family history of CAD (3.7%) Smoker (25.5%) Obesity (17.0%)	Not reported
Liang, Pang, Gao et al., 2023 [58]	China	Compare risk factors and outcomes of young STEMI patients who underwent PCI	Retrospective observational study	Total: 701 Age ≤ 45 years: 108	≤45 years	Hypertension (35.2%) Diabetes (11.1%) Family history of CAD (17.6%) Current smoker (76.9%)	Alcohol use disorder (63.9%)
Gupta, Batra, Muduli et al., 2024 [43]	India	Compare risk factor profiles and outcomes of young and older patients with MI	Retrospective observational study (registry)	Total: 5335 Age < 50 years: 1752	<50 years	Hypertension (18.5%) Diabetes (16.0%) Previous MI (7.4%) Former smoker (4.7%) Current smoker (53.5%) Obesity (11.3%)	Alcohol use daily (13.5%) Alcohol use some days (14.0%) Cancer (0.2%)
Chachar, Noor, AlAnsari et al., 2024 [44]	Bahrain	Compare clinical characteristics and outcomes of young and older patients with STEMI	Retrospective observational study	Total: 510 Age < 45 years: 95	≤45 years	Hypertension (28.4%) Diabetes (31.6%) Dyslipidaemia (33.7%) Coronary artery disease (4.2%) Family history of CAD (14.7%) Smoker (57.9%)	Not reported
King, Patel, Arora et al., 2024 [55]	USA	Compare risk factors, use of revascularisation, and outcomes in young STEMI patients	Retrospective observational study	Total: 58,083 Age < 50 years: 8494	<50 years	Hypertension (53.3%) Diabetes (25.3%) Dyslipidaemia (53.5%) Current smoker (52.2%) Obesity (20.4%)	Alcohol use disorder (5.2%) Cocaine use (3.5%) Cannabis use (5.3%) Opioid use disorder (1.3%) Malignancy (2.0%) Autoimmune inflammatory disease (1.0%) Major psychiatric disorder (14.2%)
Shamaki, Safiriyu, Antia et al., 2024 [93]	USA	Compare prevalence, predictors, and outcomes of young STEMI patients without traditional risk factors	Retrospective cohort study	Total: 41,990	≤45 years	Hypertension (61.9%) Diabetes (28.4%) Dyslipidaemia (60.4%) Smoker (67.5%) Obesity reported to be comparatively higher	Alcohol misuse and depression reported to be comparatively higher
Juan-Salvadores, Olivas-Medina, de la Torre Fonseca et al., 2024 [94]	Portugal	Compare clinical features and long term outcomes in young patients < 35 years with CAD versus older patients	Case–control study	Total: 19,321 Age ≤ 40 years: 408 Age ≤ 35 years: 109	≤40 years	Hypertension (21.6%) Diabetes (7.6%) Dyslipidaemia (53.2%) Family history of CAD (27.9%) Obesity (36.3%)	Illicit drugs and alcohol use (18.9%, 33.6% in ≤35 years) Depression (8.9%)
